# Gene losses may contribute to subterranean adaptations in naked mole-rat and blind mole-rat

**DOI:** 10.1186/s12915-022-01243-0

**Published:** 2022-02-17

**Authors:** Zhizhong Zheng, Rong Hua, Guoqiang Xu, Hui Yang, Peng Shi

**Affiliations:** 1 State Key Laboratory of Genetic Resources and Evolution, Kunming Institute of Zoology, Chinese Academy of Sciences, 650223 Kunming, China; 2 Kunming College of Life Science, University of Chinese Academy of Sciences, Kunming, 650204 China; 3 Jiangsu Key Laboratory of Neuropsychiatric Diseases and College of Pharmaceutical Sciences, Soochow University, Suzhou, 215123 China; 4 Joint Laboratory of Animal Models for Human Diseases and Drug Development, Soochow University and Kunming Institute of Zoology, Chinese Academy of Sciences, Kunming, 650223 China; 5 Center for Excellence in Animal Evolution and Genetics, Chinese Academy of Sciences, Kunming, 650223 China; 6 School of Future Technology, University of Chinese Academy of Sciences, Beijing, 101408 China

**Keywords:** Gene losses, Naked mole-rat, Blind mole-rat, Subterranean adaptations, Pseudogenes

## Abstract

**Background:**

Naked mole-rats (*Heterocephalus glaber*, NMRs) and blind mole-rats (*Spalax galili*, BMRs) are representative subterranean rodents that have evolved many extraordinary traits, including hypoxia tolerance, longevity, and cancer resistance. Although multiple candidate loci responsible for these traits have been uncovered by genomic studies, many of them are limited to functional changes to amino acid sequence and little is known about the contributions of other genetic events. To address this issue, we focused on gene losses (unitary pseudogenes) and systematically analyzed gene losses in NMRs and BMRs, aiming to elucidate the potential roles of pseudogenes in their adaptation to subterranean lifestyle.

**Results:**

We obtained the pseudogene repertoires in NMRs and BMRs, as well as their respective aboveground relatives, guinea pigs and rats, on a genome-wide scale. As a result, 167, 139, 341, and 112 pseudogenes were identified in NMRs, BMRs, guinea pigs, and rats, respectively. Functional enrichment analysis identified 4 shared and 2 species-specific enriched functional groups (EFGs) in subterranean lineages. Notably, the pseudogenes in these EFGs might be associated with either regressive (e.g., visual system) or adaptive (e.g., altered DNA damage response) traits. In addition, several pseudogenes including *TNNI3K* and *PDE5A* might be associated with specific cardiac features observed in subterranean lineages. Interestingly, we observed 20 convergent gene losses in NMRs and BMRs. Given that the functional investigations of these genes are generally scarce, we provided functional evidence that independent loss of *TRIM17* in NMRs and BMRs might be beneficial for neuronal survival under hypoxia, supporting the positive role of eliminating *TRIM17* function in hypoxia adaptation. Our results also suggested that pseudogenes, together with positively selected genes, reinforced subterranean adaptations cooperatively.

**Conclusions:**

Our study provides new insights into the molecular underpinnings of subterranean adaptations and highlights the importance of gene losses in mammalian evolution.

**Supplementary Information:**

The online version contains supplementary material available at 10.1186/s12915-022-01243-0.

## Background

Subterranean environment provides mammalian dwellers with highly stressful living conditions that are characterized mainly by extreme hypoxia/hypercapnia, darkness, and food scarcity. Over the course of their evolution for millions of years, subterranean mammals have evolved exquisite physiological and morphological modifications to cope with these challenges [1]. Excellent examples of subterranean adaptation were found in naked mole-rats (*Heterocephalus glaber*, NMRs) [2–8] and the blind mole-rats (*Spalax galili*, BMRs) [9–15]. NMRs and BMRs are phylogenetically distantly related subterranean rodents that evolved similar morphological and extraordinary physiological traits. To live in the burrow system, NMRs and BMRs evolved cylinder-shaped bodies, shortened limbs, strong claws, elongated incisors, and degenerated visual system [1]. They are highly tolerant to tissue hypoxia, resistant to oxidative stress induced by oxygen deficiency and insensitive to acid induced by hypercapnia [4, 5, 7, 11, 15]. In addition, they are both extremely long-lived rodents that live more than 20 years and are able to suppress spontaneous and experimentally induced tumorigenesis [3, 6, 14], even though cases of cancer were reported recently in zoo-housed NMRs [16]. They also evolved several species-specific traits, for example, eusociality [17], poikilothermy [18], and lack of a circadian sleep rhythm in NMRs [19]; enlarged brain structure related to sensing and orientation [20, 21], and increased muscle capillary density [22] in BMRs. These traits aroused general interests among researchers working on various fields such as evolutionary biology, genetics, aging, and cancer research. Understanding the molecular basis of extraordinary traits in NMRs and BMRs may help us address some of the most challenging questions in biology and medicine, including adaptations to extreme environments, mechanisms of hypoxia tolerance, mechanisms of aging, and cancer resistance.

Genetic and genomic studies in NMRs and BMRs have uncovered the molecular basis of some extraordinary traits. For examples, genome sequencing revealed that the amino acid substitutions in NMR UCP1 protein are associated with its unique thermoregulation, and fast evolution of BMR embryonic hemoglobin γ gene may contribute to hypoxia adaptation [4, 15]. Comparative genomics has uncovered hundreds of positively selected genes (PSGs) and convergently evolved genes that may contribute to phenotypic evolution in NMRs and BMRs [23–25], including the convergence on the *SCN9A* gene (encoding Na_v_1.7), which leads to the repeatedly evolved acid insensitivity [15, 26]. However, it should be noted that the positive selection and convergence rarely occurred in the same gene across subterranean lineages and that evolutionary changes in different genes might produce similar phenotypes [25]. In addition, transcriptome analysis identified many differentially expressed genes that are associated with longevity, hypoxia, and hypercapnia tolerance [4, 15, 27–29]. For example, two p53 target genes, *CYTC* and *CASP9*, are downregulated in BMRs under hypoxia, which may contribute to cellular protection against hypoxia-induced apoptosis [15]. *SMAD3* gene is also upregulated in an aging NMR brain, which may slow down the growth of cancer cells and contribute to cancer resistance [4]. Even though these studies identified many candidate loci that are potentially responsible for certain traits, functional assays are largely absent to validate their contributions. Moreover, most of these studies were focusing on the functional modifications of protein-coding genes and many traits cannot be explained adequately. In this regard, it is important to assess the contributions of other genetic events to subterranean adaptations.

In addition to functional modifications of protein-coding genes, other types of genetic changes can contribute to phenotypic evolution, such as pseudogenes (gene losses) [30], regulatory element mutations [31], and epigenetic modifications [32]. Gene losses, which are usually associated with regressive evolution, were recently shown to play roles in adaptive phenotypic evolution more than we expected. Examples of adaptive gene losses include: (i) in cetaceans, losses of *DSG4*, *DSC1*, *TGM5*, and *GSDMA* may contribute to the evolution of an epidermis morphology suitable for aquatic environment, and loss of *POLM* may contribute to the improved tolerance of oxidative DNA lesions [33–36]; (ii) in fruit bats, losses of *FAM3B* and *FFAR3* may help them adapt to sugar-rich diet by increasing insulin secretion and insulin sensitivity [33]; (iii) repeated losses of *PNLIPRP1* in herbivores may increase their capacity of fat storage, which is beneficial given that herbivores usually consume fat-poor diet [37]. These observations are in consistence with the “less is more” hypothesis [38]. In terms of the subterranean mammals, however, almost all current studies addressing the role of gene losses in phenotypic evolution focused on eye degeneration [4, 15, 29, 39–41], while the contribution of gene losses to other subterranean adaptations has yet to be investigated.

In this study, to assess the contribution of gene losses to subterranean adaptations, we systematically identified gene losses in NMRs and BMRs, as well as in their terrestrial relatives, guinea pigs, and rats, which were used as aboveground controls. Comparison of gene losses between subterranean and superterranean lineages identified multiple pseudogenes that might be associated with specific subterranean physiological traits including an altered DNA damage response and a specialized cardiovascular system. In addition, we provide functional evidence for the contribution of eliminating *TRIM17*, a gene independently lost from NMRs and BMRs, to hypoxia adaptation. Thus, our work provides new insights into the molecular mechanisms of adaptations to subterranean lifestyle in both NMRs and BMRs.

## Results

### Compositions of the pseudogene repertoires

**Fig. 1:**
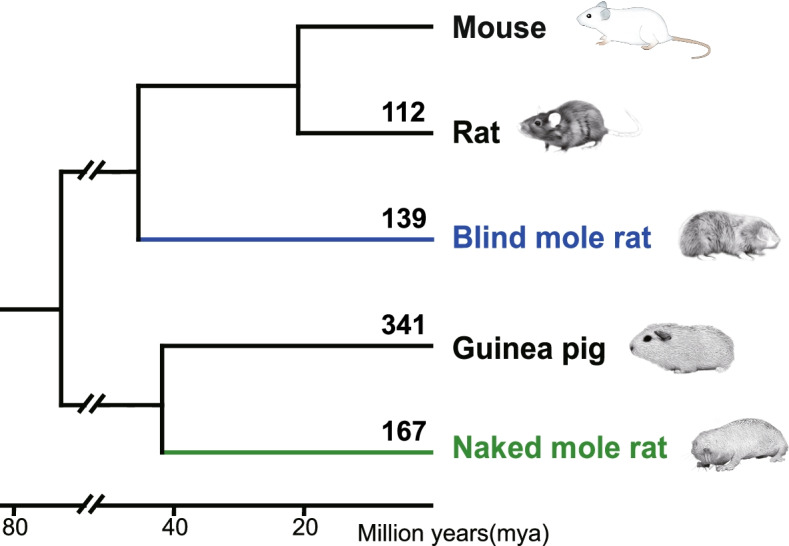
Gene losses identified in each species. The numbers of gene losses were labelled above each branch of the phylogenetic tree of species investigated

We first identified species-specific gene losses (unitary pseudogenes) in NMRs, BMRs, guinea pigs, and rats by mapping a complete mouse protein set to their genomic sequences, followed by disablement (stop codons and frameshifts) detection and filtering by rigorous criteria (Additional file 1: Fig. S1; see "Methods"). The mouse genome was used as reference because mouse is the phylogenetically closest to NMRs and BMRs with high-quality genome assembly and annotations available at present, which would mostly minimize the effects caused by phylogenetic distance. Several factors affect the validity of detected gene losses including the misjudgment of orthologous sequences, genetic redundancy [42], and the false positives introduced by misjudgment of disruptive mutations, genome sequencing/assembly errors, and annotation errors in reference genome. To address these issues and to obtain high-confidence gene losses for each species, we implemented a series of filtering steps (Additional file 1: Fig. S1; see "Methods" for details). Briefly, we first established the unitary status of each putative pseudogenic loci by (i) removing loci hit by predictively annotated proteins, intronless cDNA/expressed sequences, or genes belonging to large gene families such as olfactory receptors, zinc finger proteins, and vomeronasal receptors; (ii) removing genes with highly similar sequences at more than one locus in the genome; (iii) removing genes without conserved genomic position between query species and mouse. Secondly, we removed false disruptive mutations generated by (i) GeneWise alignment errors; (ii) the sequencing and assembly errors; (iii) potential annotation errors that resulted from the reference proteins with low transcript supporting level, or from downstream compensatory mutations that rescues the ORF disruption. As a result, 211, 211, 617, and 389 high-confidence unitary pseudogenes were identified from the NMRs, BMRs, guinea pigs, and rats, respectively. To make our results more conservative, we further removed those pseudogenes without signatures of relaxed selection (Additional file 1: Fig. S1; see "Methods") because gene dispensability and the resulting relaxed selective constraint is considered the hallmark of gene losses [30]. Finally, we obtained 167, 139, 341, and 112 gene losses in NMRs, BMRs, guinea pigs, and rats, respectively (Fig. 1, Table 1 and Additional file 2: Table S1). In order to assess the validity of our methodology, we further validated our filtering procedures with BUSCO gene sets, which are generally assumed to be conserved and universally presented in most species [43]. Using 9150 mammalian conserved BUSCO genes, we identified 20 in BMRs and 22 in NMRs as pseudogenes, suggesting ~ 0.24% putative false positive ratio, which is comparable to previous pseudogene screening studies (e.g., < 0.3% [33]). The use of 13,650 glires conserved genes showed similar results (0.30% = 41/13,650 in BMRs and 0.31% = 42/13,650 in NMRs, respectively). Thus, our pipeline produces minimal false positives.

**Table 1:** Statistics of each step of identifying gene loss events in NMRs, BMRs, guinea pigs, and rats

Species	Overlapping best hits	Putative pseudogenic loci	Unitary pseudogenic loci	False positives removed	Selective constraints relaxed
NMRs	14,254	1362	883	211	167
BMRs	16,546	1609	1023	211	139
Guinean pigs	15,969	2695	2217	617^a^	341
Rats	17,396	2821	1701	389^a^	112

^a^Sequencing errors were not examined

### Functional bias and convergence of gene losses in subterranean lineages

**Fig. 2:**
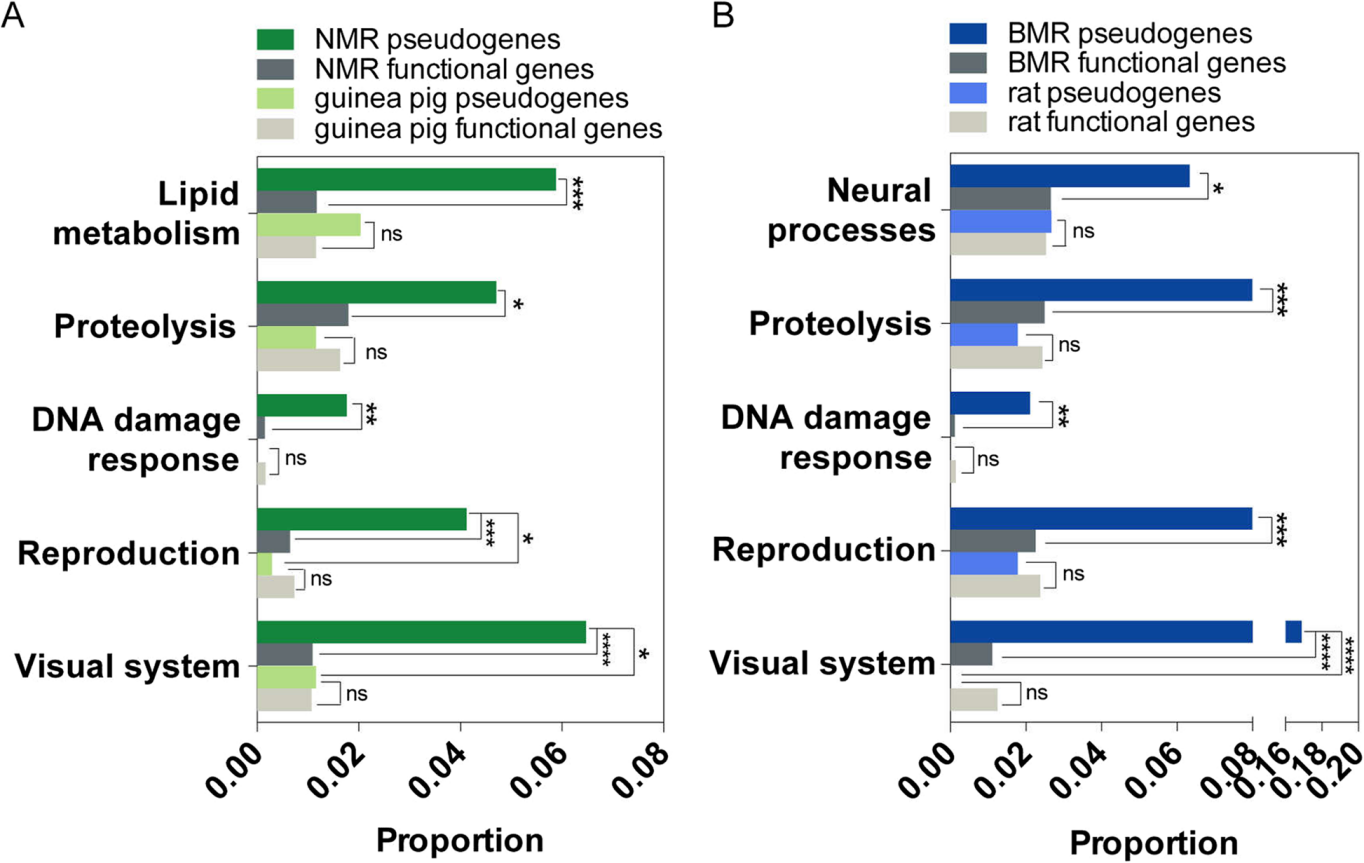
Functional groups enrichment analysis. Lineage-specific and shared EFGs identified in NMR (**A**) and BMR (**B**) pseudogenes. The proportions of pseudogenes and functional genes background from each species in each EFG are shown and proportional differences in each EFG were tested with Fisher’s exact test. *P < 0.05, **P < 0.01, ***P < 0.001, ****P < 0.0001

Next, we examined what kinds of genes were preferentially lost in NMRs and BMRs, which are more likely to be associated with subterranean adaptations. Using mouse orthologs as surrogates, we firstly obtained all associated GO terms for all pseudogenes in each species. Functionally cross-related GO terms were clustered into functional groups, and a subset of GO terms from each group that are overrepresented in subterranean lineage pseudogenes compared to subterranean lineage non-pseudogenes or control lineage pseudogenes (NMRs *vs*. guinea pigs and BMRs *vs*. rats) were identified as an enriched functional group (EFGs) (see "Methods"). Among the 24 clustered functional groups, a total of 10 EFGs were identified, with 5 in the NMR pseudogenes and 5 in the BMR pseudogenes (Fig. 2 and Additional file 3: Table S2). For each EFG, the proportion of pseudogenes from the subterranean lineage is significantly higher than that of pseudogenes from the corresponding control lineage and/or the proportion of functional genes from its own genome (Fig. 2 and Additional file 3: Table S2), indicating the functional loss of genes in these EFGs likely to be related to the subterranean lifestyle. Genes in two EFGs might be associated with species-specific traits. For example, the EFG for “lipid metabolism” might be associated with the alternating membrane phospholipid composition in NMRs [44]. The EFG “neural processes” might be associated with the enlarged brain volume in BMRs [20, 21]. In contrast, four EFGs, namely, those for the “visual system,” “reproduction,” “DNA damage response,” and “proteolysis,” were shared between the pseudogene lists from the two subterranean species. These repeatedly observed EFGs support a notion that subterranean adaptations are relatively restricted to where certain physiological process was recurrently modified to cope with stressful environments.

**Fig. 3:**
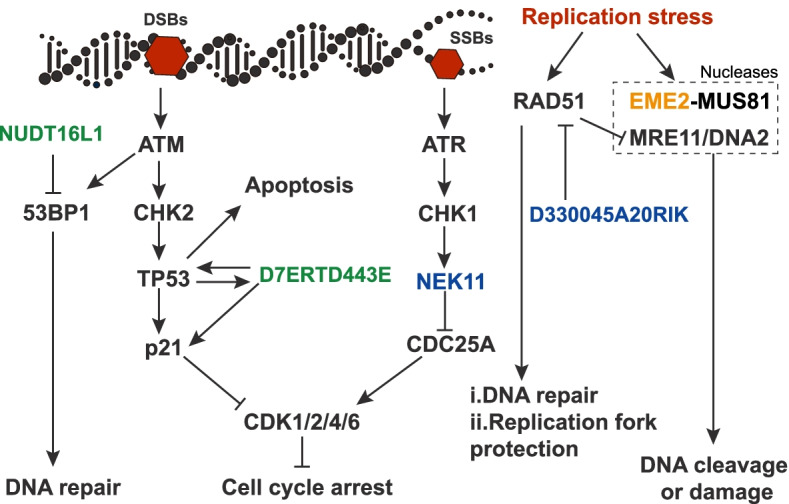
Functional convergences of gene losses between NMRs and BMRs in DDR pathway. A part of DNA damage response pathway is represented. Genes highlighted in green and blue are gene losses from NMRs and BMRs, respectively, and the gene highlighted in orange indicates shared gene loss between NMRs and BMRs

The hypoxic and hypercapnic burrow environments cause a significant oxidative stress, which can cause DNA damage, especially DNA double-stranded breaks (DSBs). We observed significant enrichments of NMR and BMR pseudogenes in “DNA damage response” EFG when compared to their own functional gene background (FDR corrected *P* = 0.0081 for NMRs and *P* = 0.0024 for BMRs, Fisher’s exact test) (Additional file 3: Table S2), while guinea pig and rat pseudogenes show no significance compared to their respective functional gene background (Additional file 3: Table S2). The genes in this EFG involve DNA damage checkpoint and DNA repair regulation, including *D7ERTD443*E, *NUDT16L1*, and *EME2* in NMRs and *NEK11*, *D330045A20RIK*, and *EME2* in BMRs (Fig. 3 and Additional file 3: Table S2 and Additional file 4: Table S3). Among them, *D7ERTD443E* (*FATS*) and *NEK11* are checkpoint related genes that affect cell cycles and apoptosis by regulating p53/p21 and CDC25A, respectively [45–48]. *NUDT16L1*(*TIRR*) and *D330045A20RIK* (*RADX*) are negative DNA repair regulators [49, 50]. *EME2* is an endonuclease component gene that is capable of causing DNA damage under replication stress [51–53] (Fig. 3). Thus, the functional loss of these DNA damage response (DDR)-related genes in NMRs and BMRs might enhance DNA repair and reduce stress-induced apoptosis, which is in consistence with the similar direction of alteration of DNA damage response observed in NMRs and BMRs [8, 54, 55]. Therefore, these convergent DDR-related gene losses are probably adaptive in response to stressful subterranean environments.

Interestingly, we also noticed that some pseudogenes in a functional group named “cardiovascular system” also show a similar pattern of functional convergence, although no significant enrichments were observed (Additional file 3: Table S2). For example, genes involved in blood pressure and heart rate regulation were detected as pseudogenes in NMRs (*TNNI3K* [56] and *RNPEP*) and BMRs (*PDE5A*) (Additional file 4: Table S3). Inhibition of *RNPEP* was shown to suppress hypertension in spontaneously hypertensive rats [57], and inhibition of *TNNI3K* and *PDE5A* was shown to be associated with suppressed contractility [58, 59]. Even though the cardiomyocyte contractility in BMRs has yet to be determined, the heart rate of BMRs was observed to be lower than rat under both normoxia and hypoxia conditions [60], which might be caused by weakened cardiac contractility. Therefore, functional convergence among these pseudogenes might be associated with the low blood pressure in NMRs and low heart rate in BMRs that caused by changes in cardiac contraction [22, 60–62]. In addition, inhibition of *TNNI3K* or *PDE5A* was shown to be cardioprotective against several cardiomyopathies [63–67]. Moreover, *TNNI3K* inhibition was also shown to increase the frequency of the mononuclear diploid cardiomyocyte (MNDCM) population and to elevate cardiomyocyte proliferation after injury [56, 68]. Taken together, the association of distinct pseudogenes with convergent adaptive traits highly suggests that gene losses could play a positive role in subterranean adaptations.

### Potential roles of convergent gene losses (CGLs) in subterranean adaptations

**Fig. 4:**
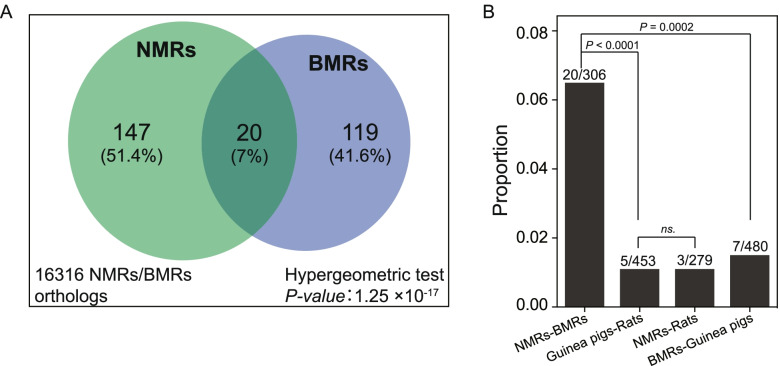
Convergent gene losses in species pairs from similar and distinct environments. **A** Venn diagram showing the one-to-one orthologous genes (large box) and overlapping pseudogenes in NMRs-BMRs pair. The numbers in parentheses represent the percentages of each category in the total number of pseudogenes in NMRs and BMRs (286 in total). *P*-value from a hypergeometric test indicates that gene losses in NMRs and BMRs are not independent from each other. **B** Proportional difference of overlapping pseudogenes among 4 species pairs including NMRs-BMRs, guinea pigs-rats, NMRs-rats, and BMRs-guinea pigs. The numbers of overlapping and total pseudogene from each pair are labelled above the bars. *P*-values are from two-tailed Fisher’s exact test

It has been shown recently that CGLs contribute to repeatedly evolved adaptations [33, 37, 69]. To reveal subterranean adaptations that were contributed by CGLs, we compared the pseudogene lists from NMRs and BMRs. Twenty CGLs were observed between NMRs and BMRs (Additional file 4: Table S3 and Additional file 5: Table S4). The hypergeometric test shows that these CGLs significantly outnumbered the random expectation (1.42, *P* = 1.25 × 10^−17^, Fig. 4A). To examine the extent to which the number of CGLs is affected by similarity of ecological niches, we further paired the four species according to the similarities and differences in their living environments. Among 4 pairs of species, NMRs-BMRs (N-B) and guinea pigs-rats (G-R) live in similar environment (homotypic pair), while NMRs-rats (N-R) and BMRs-guinea pigs (B-G) live in different environment (heterotypic pair). Comparison of pseudogene lists between paired species shows that homotypic N-B pair is the most prominent with the highest number of CGLs (Fig. 4B). In contrast, the number of CGLs from homotypic G-R pair is significantly lower than N-B pair (5/453 *vs.* 20/306, *P* < 0.0001, Fisher’s exact test) (Fig. 4B). The CGLs number from the homotypic N-B pair is significantly higher than that from heterotypic B-G pair (20/306 *vs.* 7/480, *P* = 0.0002), while there is no significant difference between the CGLs number from the homotypic G-R pair and that from the heterotypic N-R pair (Fig. 4B). In line with the functional convergence among distinct genes, the CGLs between NMRs and BMRs directly reflect their similar responses to subterranean environments and likely contributed to the evolution of shared traits.

To associate overlapping pseudogenes with subterranean traits, we first leveraged knockout mouse phenotypic information (MPI) from the literature or MGI database. Degenerated visual capacity is one of the most significant traits that shared between NMRs and BMRs. We found that 5 convergent gene losses, including *GUCY2F*, *ABCB5*, *RP1L1*, *CRB1*, and *ARR3* [70], are vision related and knockout mice of each gene show visual abnormality (Additional file 6: Table S5). It is worth noting that we also identified many other vision-related genes that lost specifically in each species, including genes reported by previously studies [4, 15, 33, 39–41], and new vision-related pseudogenes, such as *CRB1* in BMRs (Additional file 7: Table S6). Thus, these gene losses collectively contribute to the eye degeneration in NMRs and BMRs. We then searched for the significant genetic associations (*P* < 1 × 10^−6^) with all overlapping genes that curated in the NHGRI-EBI GWAS catalog [71]. Interestingly, we found that variants in 5 genes, including *ABCB5*, *LETM2*, *INMT*, *RP1L1*, and *CRB1*, are significantly associated with cardiovascular traits including PR interval, pulse pressure, and systolic blood pressure [72–76] (Additional file 8: Table S7). Although further investigations are needed, these data imply contributions of losing such genes to cardiovascular adaptations in both NMRs and BMRs, especially to blood pressure control under hypoxia.

Previous functional studies also provide clues regarding the potential roles of gene losses in subterranean adaptations. For example, loss of *EME2* could probably alleviate DNA damages induced by oxidative stress under hypoxic condition [51–53]. Another identified pseudogene, *TRIM17*, is more intriguing. It has been suggested that *Trim17* is involved in trophic factor withdrawal-induced neuronal apoptosis [77]. Considering that both NMRs and BMRs are highly tolerant to hypoxia-induced brain injury [7, 13], it is possible that independent loss of *TRIM17* in NMRs and BMRs contribute to protecting neural cells against hypoxia-induced apoptosis. However, no direct links have been established between hypoxic stress and the actions of *TRIM17* in in vivo or in vitro. It is also possible that uncharacterized functions of *TRIM17* are associated with other subterranean traits. For example, *Trim17* knockout mice show physiological traits that are not involved in nerve system, including increased circulating alkaline phosphatase level (MP: 0002968) and increased circulating thyroxine level (MP: 0005477) (Additional file 6: Table S5). Therefore, functional investigations for these less well-characterized genes would provide insights into the roles of their functional losses in subterranean adaptations.

### Functional loss of TRIM17 contributes to neuroprotection under hypoxia in vitro

**Fig. 5:**
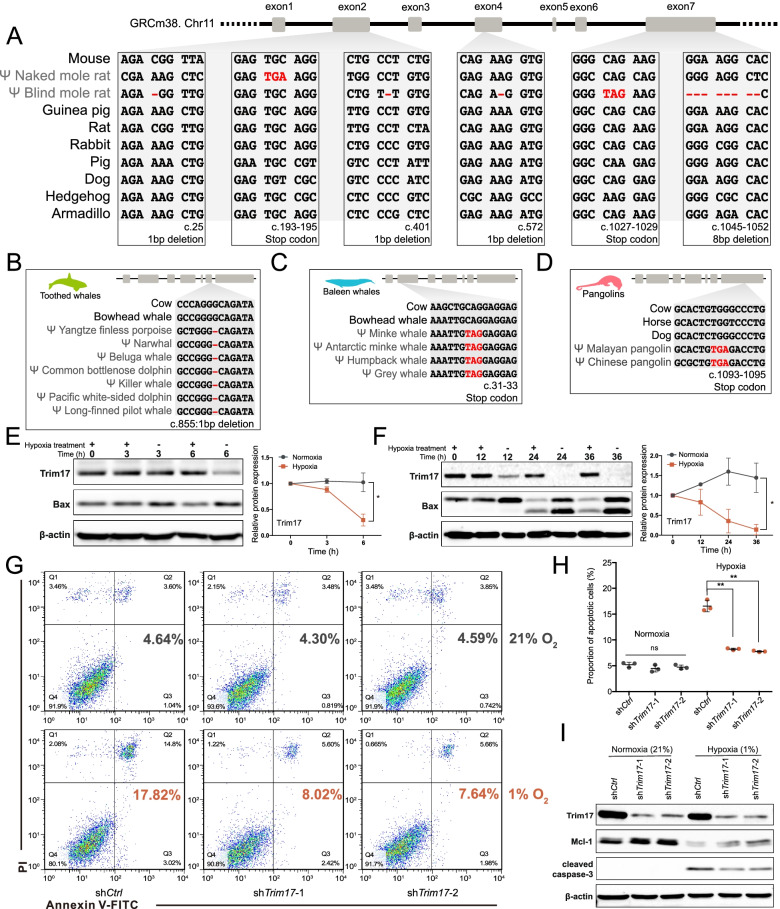
Functional tests of *TRIM17* for its role in hypoxia adaptation. **A** Coding sequence alignment of *TRIM17* orthologous genes from mouse, NMR, BMR, and 7 other mammals. One stop codon in NMRs and one stop codon/four frameshifts in BMRs were observed that resulted in disrupted coding potential of *TRIM17* gene. **B–D** Examples of inactivating mutations in toothed whales (**B**), baleen whales (**C**), and pangolins (**D**). The positions of all mutations shown in this figure are labelled under the corresponding alignment using mouse Trim17 coding sequence as reference. **E–F** Immunoblot analyses for Trim17 protein level changes during hypoxia treatments in primary cortical neurons (**E**) and N2a cell lines (**F**). “+” indicates hypoxia treatment and “−” indicates normoxia treatment. Quantification and relative fold change analysis of protein level showed a significant decrease of Trim17 protein induced by hypoxia in both primary cortical neurons and N2a cells. Data from three independent assays were used to perform paired two-tailed *t*-test, *P < 0.05. **G–I** Knockdown of *Trim17* expression by shRNAs (sh*Trim17*-1 and sh*Trim17*-2) provided neuroprotection under hypoxia (1% O_2_) compared to control (sh*Ctrl*). Apoptotic cells were detected by staining cells with PI and Annexin V-FITC followed by flowmetry analysis. Cells with *Trim17* knockdown showed significantly lower apoptosis rate under hypoxia compared to control, while no significant changes were observed under nomorxia (**G, H**), paired two-tail *t*-test, *P < 0.05, **P < 0.01. Immunoblot analysis for corresponding cells showed elevated Mcl-1 protein level and decreased activated caspase-3 protein level under hypoxia in cells with *Trim17* knockdown (**I**)

We hence chose *TRIM17* to perform functional survey to examine whether loss of *TRIM17* is involved in neuronal protection under hypoxia, which is a critical theme in hypoxic environment adaptation [78]. Disruptive mutations were confirmed by manual inspection of the alignment, showing that *TRIM17* is inactivated by one premature stop codon in NMRs and 1 stop codon/4 indels in BMRs (Fig. 5A). The RING finger domain of TRIM17 is indispensable for its E3 ligase function [77] but is disrupted in both species, hinting the functional loss of this gene. We also examined the frequency of disruptive mutations in 11 BMR individuals [79], and no segregation was found. In NMRs, the premature stop codon was also verified by the available transcriptomic reads and genomic sequence of another individual (Additional file 4: Table S3). Furthermore, *TRIM17* shows signature of relaxed selective constraint in both species, though not significant (NMRs: *k* = 0.69, *P* = 0.1338; BMRs: *k* = 0.72, *P* = 0.1214) (Additional file 2: Table S1). These results together confirmed the inactivation of *TRIM17* in both NMRs and BMRs. Interestingly, we noticed that *TRIM17* was independently lost at least two times in cetaceans, with a shared 1 bp deletion in toothed whales (Monodontidae, Phocoenidae, and Delphinidae) (Fig. 5B) and a shared premature stop codon in baleen whales (Balaenopteridae) (Fig. 5C), in consistent with a previous report [33]. Inactivating mutations were also detected in several species in other families including right whale (*Eubalaena japonica*), pygmy sperm whale (*Kogia breviceps*), and boutu (*Inia geoffrensis*) (Additional file 1: Fig. S2). Moreover, *TRIM17* was also lost in two pangolins (*Manis pentadactyla* and *Manis javanica*) due to a shared premature stop codon (Fig. 5D) [33]. Like NMRs and BMRs, cetacean and pangolins experience hypoxic stress during their lifetime [78, 80, 81]. Therefore, independent losses of *TRIM17* in distinct hypoxia-tolerant taxa suggest a relevance of *TRIM17* losses in hypoxia adaptation.

To illustrate this idea, we next examined the changes in Trim17 expression in response to hypoxia in both Neuro-2a (N2a) cells and mouse primary cortical neurons. An immunoblot analysis showed that in contrast to trophic factor withdrawal, the Trim17 protein level was significantly decreased in both N2a cells and primary neurons after treating with hypoxic stress (1% O_2_) (Fig. 5E–F and Additional file 1: Fig. S3), suggesting a hypoxia-responsive regulatory mechanism for Trim17 protein. We further tested whether Trim17 knockdown confers a neuroprotective effect under hypoxia. N2a cell lines stably expressing shRNAs that targeted Trim17 and control shRNA were constructed and treated under hypoxic condition (1% O_2_) for 48 h, followed by an apoptosis analysis via flow cytometry. In contrast to the increased apoptotic rate in the control cells (sh*Ctrl*), the Trim17 knockdown cells (sh*Trim17*-1, 2) showed significantly reduced apoptosis under hypoxia compared with normoxia (21% O_2_) (Fig. 5G–H). As Trim17 was shown to initiate apoptosis through the proteasome-dependent degradation of Mcl-1 [82], we examined whether this is also the case under hypoxia. An immunoblot analysis revealed elevated Mcl-1 protein level and consequently decreased active caspase-3 protein under hypoxia when compared to the control (Fig. 5I and Additional file 1: Fig. S3). Taken together, these results suggested that by preventing Mcl-1 protein from degradation, eliminating Trim17 function confers neuroprotection against hypoxia. Further investigations addressing the linkages among the components of phenotype-genotype-fitness continuum would provide us a better understanding of the contribution of gene loss to animal adaptation.

### Gene losses and PSGs cooperatively contribute to subterranean adaptations

**Fig. 6:**
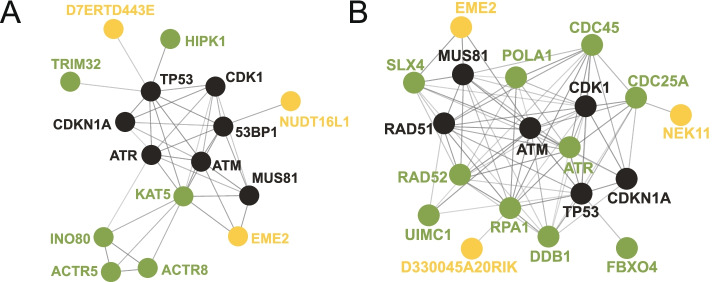
Interacting network of DDR-related proteins. Different genes with different types of genetic changes are interacting with each other in both NMRs (**A**) and BMRs (**B**). PSGs are highlighted in green and gene losses are highlighted in yellow, and core mediators of DDR are highlighted in black. Interaction information was derived from STRING database (https://string-db.org/, Version 11.0)

Complex physiological adaptations, such as modifications of DNA damage response and cardiovascular system, are expected to be involving multiple genes. We noticed that many genomic studies had uncovered PSGs that are also involved in DDR and heart functions in NMRs and BMRs [4, 15, 25, 29]. Unlike gene losses, PSGs show a signature of selection that can be easily detected, and thus could be assigned as adaptive. To examine whether PSGs also show enrichments in functional groups that similar to EFGs in pseudogenes, we collected PSGs from previous genomic studies and compiled a PSG set for NMRs [4, 29] and BMRs [15, 25], respectively (see “Methods”). Interestingly, for some of the EFGs in pseudogenes, we also observed similar functional enrichments in their respective PSG sets, including functional groups related to “DNA damage response,” “lipid metabolism,” and “neural processes” (Additional file 9: Table S8). Moreover, in consistent with the phenotypic convergence in heart contractile function, PSGs in functional group of “cardiovascular system” also show significant enrichment (Additional file 9: Table S8). In fact, several genes involved in cardiac muscle contraction in both species were under positive selection. These observations highlight the collaborative contributions of not only different genes, but also different types of genetic makeups to physiological adaptations. To illustrate this, we showed that in both NMRs and BMRs, protein-protein interaction network (STRING) involving DNA damage response contain both pseudogenes and PSGs, and both of them interact with other critical mediators such as *ATM*, *ATR*, or *TP53* (Fig. 6). Such simultaneous gain-of-function and lost-of-function changes in multiple genes among a network reflect a strong rewiring process in response to drastic environmental changes, supporting an adaptive role of gene losses during subterranean adaptations.

## Discussion

### Challenges in genome-wide identification of adaptive gene losses

In this study, we systematically identified gene losses in two well-adapted subterranean rodential lineages, NMRs and BMRs, to examine the contribution of gene losses to subterranean adaptations after they split from mouse. By far, the identification of pseudogenes remains a challenging work because many factors can affect the final repertoires, including not only the sequencing, assembling, annotating qualities, but also the reference selection. Even though several genomic studies had identified pseudogenes in BMRs and NMRs (e.g., [4, 15]), to the best of our knowledge, this is the first study to identify gene losses using mouse rather than human as reference. For this reason, the repertoires of pseudogenes identified in our study would differ from others. For example, many pseudogenes identified using human as reference were generated in the ancestor of rodents and are not included in our pseudogene list. Meanwhile, it is not possible to identify loss of rodent-specific genes in NMRs and BMRs using human reference but this could be achieved using mouse reference. Our work thus provides an opportunity to survey the lineage-specific pseudogenization within rodents. Additionally, differences in the identification strategy and filtering criteria can generate different pseudogene repertoires as well. For example, the pseudogene lists of previous studies contain not only unitary pseudogenes, but also pseudogenes with potential functionally redundant paralogs. Here, we applied stringent criteria to maximumly reduce false positives (see “Methods”), e.g., the additional application of filtering signature of relaxed selection (RELAX) provides a most conservative result. The validation using BUSCO genes proved that our pipeline produces minimal false positives, although some real pseudogenes might be neglected due to the stringent filtering criteria. For example, *RIPK3*, a critical component in the necroptosis pathway, was recently reported to be lost in NMRs [83]. Our approaches did detect the disruptive mutations in *RIPK3* sequence in both NMRs and BMRs. However, they were removed from our final list due to the poor GeneWise alignment (NMRs), or no signature of relaxed constraint (BMRs). In this way, we tried to guarantee the pseudogenes on our list are most reliable. Despite this, previously reported pseudogenes such as *FASTK* [4], *MTNR1A* [4], *MTNR1B* [4], *TNNI3K* [56], and *AWAT2* [84] in NMRs and many vision-related pseudogenes in both NMRs and BMRs (Additional file 7: Table S6) are identified in our study, validating the effectiveness of our approaches. Collectively, our results provide a new perspective on subterranean adaptation at a smaller evolutionary scale and with higher resolution and give the community more insights into the molecular changes in response to subterranean environments.

Gene losses were generally thought to be deleterious and many pseudogenes in mammalian genome are the consequences of relaxed selective constraints. It is not an easy task to claim adaptive gene losses. Population genetic analysis could provide some evidences for such adaptation. As exampled by the *CASPASE12* gene in human populations [85], the complete or nearly complete fixation of a null allele, in contrast to the high nucleotide diversity of the sequences adjacent to this gene, is the signature of positive selection and thus indicates an adaptive gene loss. However, population genetics approaches can only detect recent positive selection signatures (< 250,000 years) [86], and thus limit our capability of detecting ancient adaptive gene loss events. Unfortunately, the subterranean adaptations of both NMRs and BMRs occurred more than 30–40 Myrs ago [15], far beyond the detecting range of population genetics approaches. The sequence of ancient pseudogenes, even though they were adaptive and underwent positive selection at the early stage, usually evolves neutrally afterwards, making it difficult to detect the signal of positive selection. The direct evidence for an adaptive gene loss is the increased fitness of individuals with gene function loss compared to individuals with normal gene function in a population. However, it is a challenge to perform such tests for most of the non-model mammals. Previous genomic studies usually claim adaptive gene losses by associating them with adaptive traits, leveraging the available gene functional information or extrapolating phenotypic information from model organisms [33–37]. In this study, we also tried to link pseudogenes with adaptive traits of NMRs and BMRs based on currently available resources. We conducted genomic survey and functional group enrichment analyses to highlight several cases of gene losses to support the notion of adaptive gene losses, including genes involving DNA repair and cardiac contraction. More importantly, we tried to evidence their adaptive roles by showing the functional convergence of different gene losses in different species, in vitro functional assays of a convergent gene loss (*TRIM17*), and the interaction between pseudogenes and PSGs. Although further studies are still necessary to add more details in this process, our work provides a primary overview for the adaptive gene losses in subterranean lifestyle of NMRs and BMRs.

### The adaptive role of losing TRIM17 in subterranean mole-rats

Despite the fact that current studies generally associate pseudogenes to the adaptive traits through information integration and speculation, the experimental evidence, either in vitro or in vivo, is more persuasive. In this study, we used *TRIM17*, one of the 20 convergently lost genes, as an example to demonstrate the beneficial role of gene inactivation in the subterranean adaptation. Although *Trim17* has been reported to regulate neuronal apoptosis [77], its relationship with hypoxic insults is still not clear. For this reason, we provided functional evidence from an in vitro assay for the involvement of *Trim17* in the response to hypoxia, and demonstrated the beneficial effects of losing *Trim17* in neuroprotection under hypoxia. Therefore, by directly linking *Trim17* with hypoxia, our results support the positive roles of inactivation of *TRIM17* in both NMRs and BMRs in hypoxia tolerance during their transition to subterranean niches.

It is notable that *Trim17* is also highly expressed in mouse testis, which usually implies an important reproductive related function. Unexpectedly, the *Trim17* knockout mice show no abnormality of composition, quantity, and morphology of spermatogenic cells; the morphology and motility of the mature spermatozoa are not affected; and the fecundity of knockout males is comparable to that of the wild type [87]. This is probably due to the non-essentiality of Trim17 protein to fertility and the inactivation of Trim17 in testis might be compensated by other TRIM family members such as Trim58 or Trim69 [88]. In contrast, this compensation was not observed in brain/neuronal cells [77], in consistent with our observation of differentiated cell death between wild type and *Trim17* knockdown N2a cells (Fig. 5G, H). A plausible explanation is that subterranean NMRs and BMRs may utilize some kind of tissue-specific mechanism to maintain the beneficial neuroprotective role of inactivated TRIM17 while not to dampen the fertility. Nevertheless, further investigations are necessary to address this issue in the future.

### The interaction of PSGs and pseudogenes implies adaptive gene losses

For decades of years, the PSGs and pseudogenes are rarely discussed together, due to the traditional understanding of their distinct phenotypic effects. To the best of our knowledge, this study, for the first time, examined the interaction of PSGs and gene losses and highlighted a scenario in which PSGs and pseudogenes work together to promote adaptations. The occurrence of PSGs in a network containing pseudogenes suggests an evolution direction of the network that is not likely to be regressive, but adaptive. Therefore, this observation highly suggests the adaptive roles of gene losses in this network. Meanwhile, it also raises a question of how gene losses and PSGs work together to achieve a functional benefit. One can easily speculate that gene losses and PSGs may have similar functional effects that together result in an additive effect on a trait. For example, in the case of NMR DDR network, two genes, a PSG *TRIM32* and a pseudogene *D7ERTD443E* (*FATS*) interact directly with *TP53* (Fig. 6A). Both of them are ubiquitin ligases, but they regulate protein stability of TP53 in opposite directions, with a ubiquitination-mediated stabilization by FATS and a ubiquitination-mediated degradation by TRIM32 [48, 89]. Pseudogenization of *FATS* eliminates one layer of positive control of TP53 protein stability and alternatively facilitates negative regulation together with TRIM32, or other negative regulators. There might be other possibilities including the “sign-epistatic” interaction [90] and the compensation hypothesis that pseudogenes serve as a buffer for deleterious changes [91], although both await further investigations for verification in the future. Nevertheless, it is more likely that all these possible interactions exert their phenotypic effects simultaneously, due to the rewiring of a complex genetic network underlying physiological adaptations, especially to hypoxia. Our results highlight the complexity of underlying genetic mechanisms of subterranean adaptations, and considering the effects of different types of genetic makeups simultaneously could help us get a better understanding of adaptive evolution to subterranean niches or other extreme environments.

## Conclusions

With combination of comparative genomic approaches and in vitro experimental validations, our study, for the first time, uncovered that gene losses might contribute to the evolution of specialized physiological traits in both NMRs and BMRs, including altered DNA damage response, specialized cardiovascular system, and neuroprotection. We also highlighted that the genetic mechanisms underlying physiological adaptations to subterranean niches are complex, involving not only multiple genes but also different types of genetic makeups. Taken together, our study provides new insights into the molecular underpinnings of subterranean adaptations, highlighting the important roles of gene losses. Several pseudogenes identified in BMRs and NMRs might have implications for medical researches involving aging and longevity, neuroprotection in ischemic/hypoxic injury, and cardiovascular diseases. Moreover, our study suggests an integration of different genetic makeups in future studies and also has implications for the comprehensive understanding of subterranean adaptation.

## Methods

### Genomic data

The genome sequences of each species analyzed in this study were retrieved from the Ensembl database [92] (mouse: GRCm38.p2, release 75; rat: Rnor_6.0; guinea pig: Cavpor 3.0, release 89) and NCBI database [93] (NMR: HetGla_female_1.0; BMR: S.galili_v1.0) together with its annotated coding sequences (CDS) and protein sequences. The CDS and protein sequences from rabbit, which were used to estimate the evolutionary rate, were retrieved from the Ensembl database (OryCun2.0).

### Identification of gene losses

Basically, the gene losses in each species are defined as genes harboring ORF-disrupting mutations, including premature stop codons and/or frameshifts with intact 1:1 orthologs in the mouse genome. Orthologous relationships were first established by mapping the longest protein sequence for each mouse gene to each query species by simultaneously using BLAT [94] and genBlastA [95]. The genomic regions identified by both BLAT and genBlastA were considered as orthologous sequences and were extracted with 5000 bp downstream and upstream flanking sequences to predict the gene structure and ORF by GeneWise [96]. Alignments containing premature stop codons and/or frameshifts were identified, and the corresponding genomic regions were defined as putative pseudogenic loci. To establish the unitary status of each putative pseudogenic locus, several stringent filtering steps were executed as follows: (1) Loci hit by proteins belonging to large gene families including olfactory receptors, zinc finger protein, and vomeronasal receptors were removed because of their high sequence similarity. (2) Loci hit by predicted proteins or intronless cDNA/expressed sequences were removed because they are unlikely to be real or unitary [97]. (3) To remove the potential functional redundancy, e.g., gene duplication or retrotransposition, BLAT mapping hits from the initial step were reanalyzed to remove loci with homologous sequences across the genome. In detail, for each locus, we firstly removed hits with E-value higher than 1 × 10^−3^, and then we assembled the remaining hits into “copies” of this pseudogene along the mouse protein coordinates. Each copy includes non-overlapping hits that together cover the mouse protein as long as possible. Simply, for each “copy” with total protein length longer than 80% of the corresponding mouse protein, we considered it as a potential functional redundancy and therefore removed it. (4) We examined the conserved genomic position for each locus to ensure the orthologous relationship. Pairwise genomic alignments between mouse and each query species were downloaded from Ensembl database. For each locus, the genomic coordinates identified by us were compared to the coordinates inferred from a pairwise genomic alignment. Only pseudogenic loci with conserved genomic position were retained for further consideration. Furthermore, for genes discussed in the main text, we also manually examined the conserved gene order according to mouse genome annotation in Ensembl. To generate high-confidence pseudogene sets, false positives with disruptive mutations introduced by GeneWise, sequencing errors, or annotation errors were removed through the following steps: (a) in-house Perl script filtering in combination with manual inspection was performed to remove false positives introduced by GeneWise. Pseudogenes with a single disruptive mutation located at the end of the coding region (which resulted in truncated sequences longer than 90% of predicted intact protein [98], at the intron and exon boundaries or in regions with low similarity (30 bp up- and downstream the mutation, < 40% identity) in GeneWise alignments were removed. Pseudogenes with multiple disruptions were manually inspected to remove short or low-quality GeneWise alignments. For the genes discussed in the main text, we realigned the mutated exons on mouse genome sequence using CESAR [99] to remove spurious disruptive mutations caused by evolutionary splice site shifts. (b) To remove the sequencing and assembly errors, we retrieved the raw sequencing reads from NMRs and BMRs to check the validity of each disruption in the corresponding reads. For genes discussed in the main text, we further checked their validity using additional genomic/transcriptomic resources, i.e., raw transcriptomic reads of different tissues [100] and genomic sequences of a male individual for NMRs (HetGla_1.0) [101]; resequencing reads of 11 individuals for BMRs [102]. (c) The coding potential of the corresponding genes was examined by checking the Ensembl transcriptional support level (TSL, level 1/2) and CCDS assignment, and those satisfying any of the criteria were retained. (d) Pseudogenes with only two compensatory frameshifts were manually examined and removed because it provided little evidence for gene loss.

### Detection of relaxed selection

Genes with disruptive mutations on coding sequence do not necessarily indicate gene dispensability. Such disruptive mutations might be rare genetic variation or sequencing/assembly errors. To address this issue, we detected signature of relaxed selection for each pseudogene by RELAX [103] and using a phylogenetic tree with rabbits as the outgroup. In detail, the coding sequences (CDS) and their translations (with disruptive mutations removed) of each pseudogene were extracted from the GeneWise alignments. The protein sequences were used to identify 1:1 orthologous gene in the rest of the species for each pseudogene by reciprocal best BLAST hit (RBBH) method, and then, the results were clustered into orthologous groups. For each orthologous group, CDS were used to construct codon-based multiple sequence alignment with a phylogeny-aware alignment algorithm as implemented in the PRANK program [104]. To obtain reliable CDS alignments, we set up rigorous filtering criteria including: (i) removing gaps and ambiguous bases together with 6 bp flanking sequences with Gblocks [105] using the default parameters; (ii) removing alignment fragments with low quality (Gblocks, default parameters); and (iii) removing alignments with lengths of less than 150 bp. Alignments containing at least two sequences were assigned to a specific tree file. In each alignment, the species in which this gene loss occurred was set as “Foreground” branch and the rest of species were set as “Background” branch. Only pseudogenes with a higher dN/dS in foreground and a selection intensity parameter *k* < 1 (*k* > 1: intensified selection; *k* < 1: relaxed selection) were retained.

### Gene ontology

Orthologous mouse genes were used as surrogates to infer the functions of the pseudogenes or PSGs in corresponding species. Only pseudogenes and PSGs with orthologous relationship with mouse (established by BLAT and genBlastA mapping) were further considered. To identify the EFGs from pseudogenes, the associated GO terms for each gene were extracted from go.obo (released February 20, 2018), and the mouse gene annotation files (*Mus musculus*, released September 29, 2017) were downloaded from the Gene Ontology Consortium (http://geneontology.org/). NMR and BMR genes with GO terms involved in certain biological processes and systems were manually clustered into functional groups based on the keywords. For each functional group, we then compared the proportional differences of genes from subterranean lineage pseudogene lists, subterranean lineage non-pseudogene lists (with one2one mouse orthologs), control lineage pseudogene lists, and control lineage non-pseudogene lists (with one2one mouse orthologs). For example, a subset of GO terms with a significantly higher proportion of NMR pseudogenes compared to that of NMR non-pseudogenes and/or that of guinea pig pseudogenes was defined as an EFG. For PSGs, EFGs were defined only when significantly higher proportion of NMR (or BMR) PSGs in a subset of GO terms was observed compared to that of NMR (or BMR) non-PSG background (with one2one mouse orthologs). Fisher’s exact test was used to test the significances of differences in proportion and FDR-adjusted *P*-values for each multiple test were reported using p.adjust() function in R.

### Cell culture

Primary cortical neurons were prepared from E16-E18 embryos of Kunming mice. Specifically, embryonic cortices were dissected in cold DMEM (Gibco) supplemented with 10% fetal bovine serum (Gibco) and then digested using 2 mg/ml papain (CHI). The resulting cell suspension was centrifuged at 1500 rpm for 5 min. The cells were gently resuspended in DMEM supplemented with 10% FBS and gentamycin (50 μg/ml, Solarbio) and filtered through a 70-μm cell strainer (Corning Falcon). Then, the cells were seeded at a density of 1.2 × 10^6^ cells/ml in culture dishes coated with 20 μg/ml poly-D-lysine (Sigma). The neurons were cultured at 37 °C in a humidified incubator with 5% CO_2_/95% air, and the medium was replaced 6 h afterwards with neurobasal medium (Gibco) supplemented with B27 supplements (Gibco) and L-glutamine (Gibco). After 6 days in vitro (DIV 6), the neural cultures were subjected to hypoxia treatment and protein extraction. N2a cells were purchased from the Cell Bank of Type Culture Collection of the Chinese Academy of Sciences (Shanghai; Institution Code: CBTCCCAS) and were cultured at 37 °C in a humidified incubator with 5% CO_2_/95% air with MEM (Gibco) supplemented with 1.5 mg/ml NaHCO_3_, 0.11 mg/ml sodium pyruvate (Gibco), and 10% FBS.

### Hypoxia treatment paradigms

The hypoxia (1% oxygen concentration) for the cell culture treatment was achieved using a humidified hypoxia chamber (Proox, model C21, BioSpherix) set at 37 °C, 1% O_2_, and 5% CO_2_ and maintained with a N_2_ supply. The primary cortical neuron cultures and N2a cells (including the stable cell lines) were treated at 1% hypoxia for various periods of time. As controls, cells were cultured at 37 °C in a humidified incubator with 5% CO_2_ and 95% air.

### Immunoblot analysis

Primary cortical neuron and N2a cells (including stable cell lines) under both hypoxic and normoxic conditions were collected and lysed using RIPA buffer (Biomiga) supplemented with a protease inhibitor phenylmethanesulfonyl fluoride (PMSF) and cocktail. Immunoblotting experiments were performed according to a previous method [106, 107]. Briefly, proteins were separated by 10% SDS-PAGE gel followed by electrotransfer to PVDF membranes (Millipore). The membranes were blocked with TBST supplemented with 10% skim milk for 30 min at room temperature and further probed with primary antibodies at 4 °C overnight including Trim17 (1:500, Sigma); Mcl-1 (1:1000, CST); Bax (1:1000, CST); and cleaved caspase 3 (1:500, CST) with β-actin (1:500, GeneTex) as the loading control. The membranes were subsequently incubated with HRP-conjugated secondary antibodies, and the protein bands were detected by ECL (Thermo Scientific). The quantifications of the protein expression levels were conducted by the gray scanning of the target proteins with Image-Pro Plus 6.0 software. Three independent experiments were performed to test the expression differences.

### shRNA-lentiviral infection and apoptosis detection of N2a cells

The HIV-derived lentiviral vector pLKO.1 containing shRNAs (TRIM17 MISSION shRNA Bacterial Glycerol Stock, Sigma) that targeted Trim17 (sh*Trim17*-1: CCATCTGCCTTGACTACTTTA; shTrim17-2: CTGTTACCCAATTCCACTCTA) and control shRNA (sh*Ctrl*: GACACTGGGTGTGCCACAGTT) together with lentiviral packaging plasmids pCMVΔ8.9 and pMD2.G were co-transfected into HEK293T cells to generate lentiviruses. Supernatants containing different lentiviruses were collected 48 h and 72 h post-transfection, respectively. N2a cells were infected with supernatants in the presence of 6 μg/ml polybrene for 48 h, and 2 μg/ml puromycin was used to select the stable cell lines. Three stable cell lines expressing shRNAs were cultured simultaneously under 1% hypoxia and 21% normoxia for 48 h and collected for FITC-conjugated annexin V (BD Pharmingen) staining followed by apoptotic cell number analysis by flow cytometry according to a method described previously [108]. Three independent experiments were performed for apoptosis detection to test the differences.

### Statistical analysis

Two-tailed Fisher’s exact tests were used to detect the proportional differences in pseudogenes for each functional group. Paired *t*-tests were used to analyze differences in Trim17 protein levels and apoptosis rates. Hypergeometric tests (http://systems.crump.ucla.edu/hypergeometric/index.php) were used to assess the significance of the overlapping pseudogenes.

## Supplementary Information

**Additional file 1: Fig. S1.** Flowchart for identifying gene loss events in NMRs, BMRs, guinea pigs and rats. **Fig. S2.** Inactivating mutations of *TRIM17* in another three cetaceans. **Fig. S3.** Original western blot images.

**Additional file 2: Table S1.** Gene losses identified in each species.

**Additional file 3: Table S2.** Results of functional group enrichment analysis.

**Additional file 4: Table S3.** Mutations of pseudogenes discussed in the main text.

**Additional file 5: Table S4.** Independent gene losses between NMRs and BMRs.

**Additional file 6: Table S5.** MGI MP information for overlapping pseudogenes.

**Additional file 7: Table S6.** Vision related pseudogenes.

**Additional file 8: Table S7.** Genetic associations of pseudogenes with cardiovascular traits.

**Additional file 9: Table S8.** Functional group enrichment analysis in PSGs in NMRs and BMRs.

## Data Availability

The datasets supporting the conclusions of this article are included within the article and its additional files. The Perl scripts used for pseudogene screening and identification are available on GitHub (https://github.com/zzheng24/Gene-losses).

## Abbreviations

BMRs: Blind mole-rats
CDS: Coding sequences
CGLs: Convergent gene losses
DDR: DNA damage response
DSBs: Double-stranded breaks
EBI: The European Bioinformatics Institute
EFGs: Enriched functional groups
GO: Gene Ontology
GWAS: Genome-Wide Association Studies
HRP: Horseradish peroxidase
MGI: Mouse Genome Informatics
MNDCM: Mononuclear diploid cardiomyocyte
MPI: Mice phenotypic information
NHGRI: National Human Genome Research Institute
NMRs: Naked mole-rats
ORF: Open reading frame
PSGs: Positively selected genes
