# Supplementary figures and images for: Gene losses may contribute to subterranean adaptations in naked mole-rat and blind mole-rat
Source: BMC Biol. 2022 Feb 17;20:44. doi: 10.1186/s12915-022-01243-0 (PMC8851862; doi:10.1186/s12915-022-01243-0)

**Fig. S1**

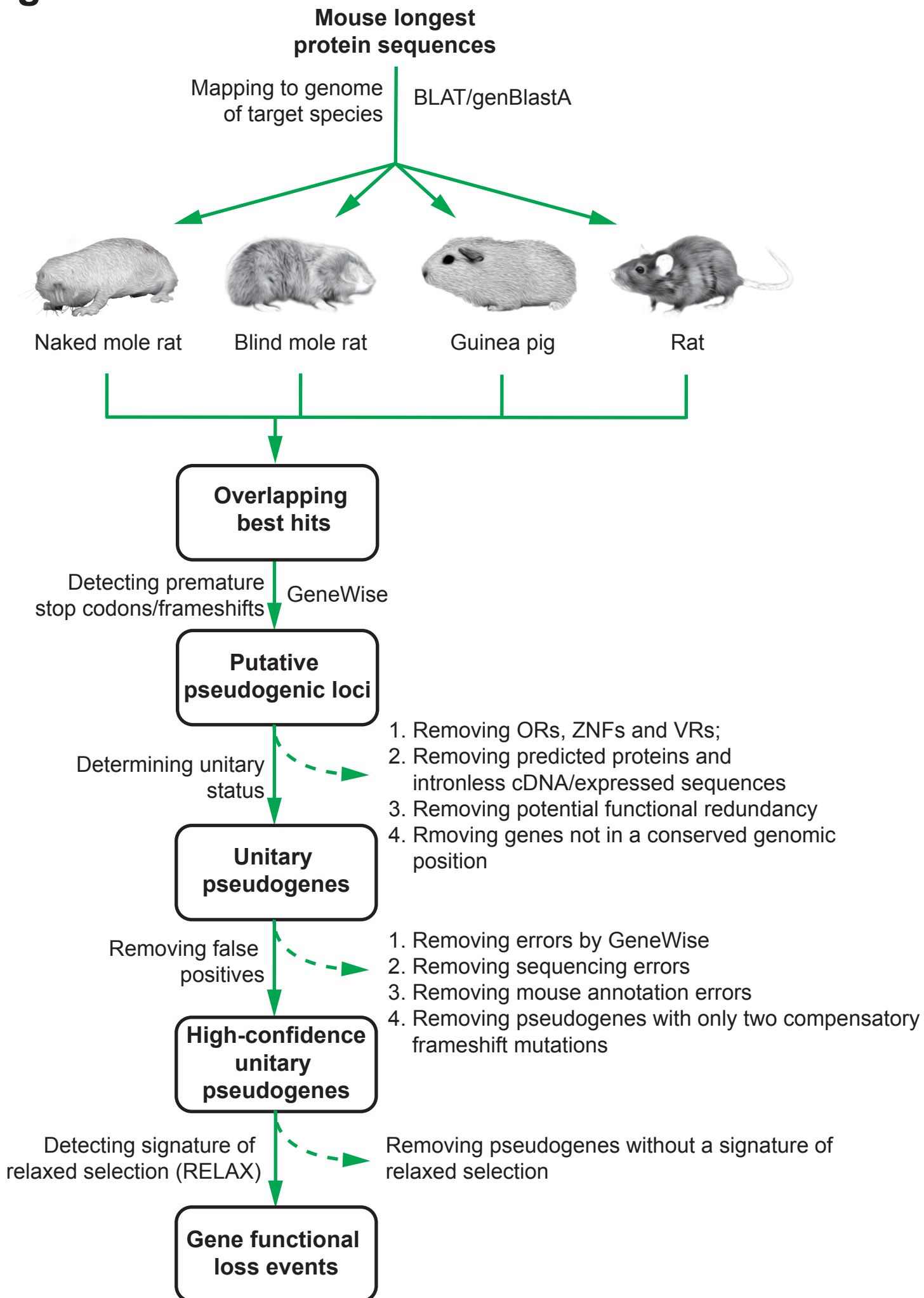

Fig. S2

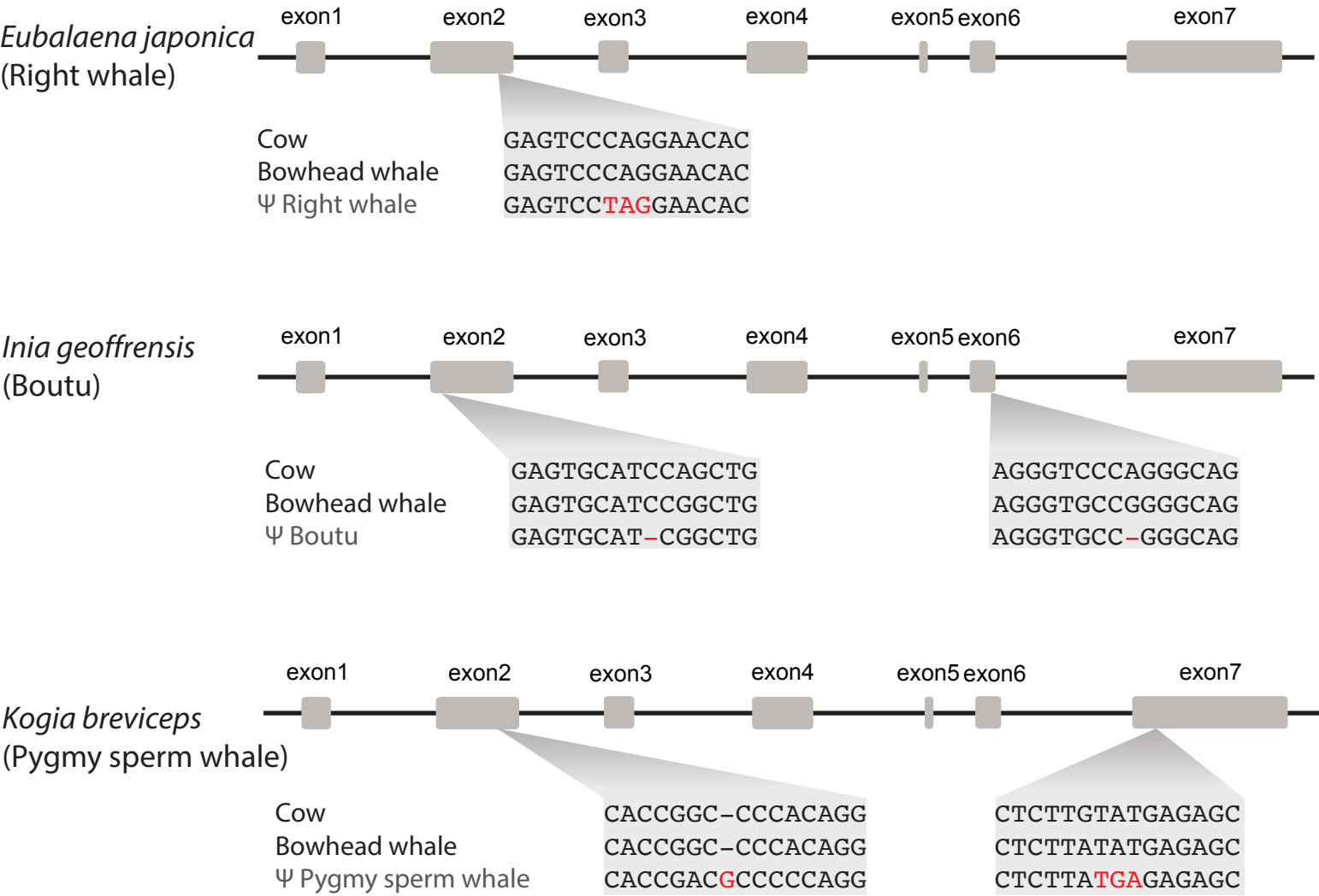

# Fig. S3

## Original western blots for Figure 5

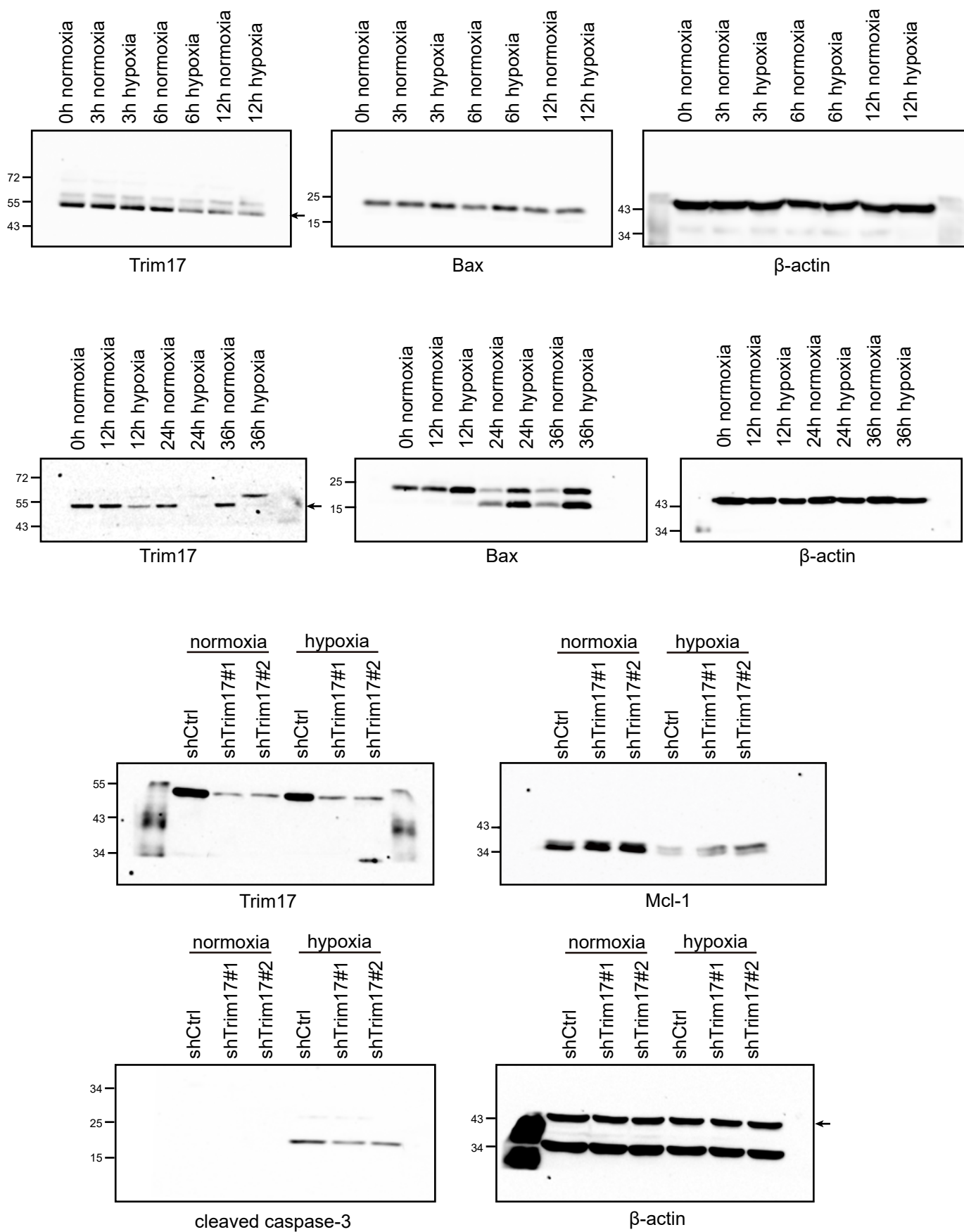

Supplement: Supplementary file 1 — Additional file 1: Fig. S1. Flowchart for identifying gene loss events in NMRs, BMRs, guinea pigs and rats. Fig. S2. Inactivating mutations of TRIM17 in another three cetaceans. Fig. S3. Original western blot images. [file 12915_2022_1243_MOESM1_ESM.pdf]
